# A conversation analytical study of call openings in Emergency Medical Service calls where the patient is at imminent risk of out-of-hospital cardiac arrest

**DOI:** 10.1016/j.resplu.2024.100706

**Published:** 2024-07-05

**Authors:** Kim Kirby, Sarah Voss, Jonathan Benger, Rebecca K. Barnes

**Affiliations:** a University of the West of England, Bristol, United Kingdom; b South Western Ambulance Service NHS Foundation Trust, United Kingdom; c Nuffield Department of Primary Care Health Sciences, University of Oxford, United Kingdom

**Keywords:** Emergency Medical Services, Call-taker, Emergency Medical Dispatch, Conversation Analysis, Out-of-Hospital Cardiac Arrest

## Abstract

**Background:**

The Chain of Survival identifies the importance of early recognition of patients who are at imminent risk of out-of-hospital cardiac arrest. This research investigated the interaction between callers and call-takers during calls to the Emergency Medical Service; it specifically focussed on patients who were alive at the initiation of the EMS call, but who subsequently deteriorated into out-of-hospital cardiac arrest during the prehospital phase of care (i.e., before arrival at hospital).

**Methods:**

Conversation-analytic methods were used to examine the call openings of 38 Emergency Medical Service calls for patients who were at imminent risk of out-of-hospital cardiac arrest. Call openings centred on pre-triage questions designed to rapidly identify patients who are either in out-of-hospital cardiac arrest, or who are at imminent risk of out-of-hospital cardiac arrest.

**Results:**

Emergency Medical Service call openings did not facilitate efficient and accurate triage, thus delaying the identification of critically unwell patients by call-takers. In 50% of call openings, the caller wanted to give the reason for the call during the pre-triage questions. The caller and call-takers orientate to different agendas causing delays to call progression and risking information loss that impacts on effective call triage.

**Conclusions:**

The design of the Emergency Medical Service call opening can cause interactional trouble, thus impacting on call progression and risking critical information loss. Modifications to the Emergency Medical Service call opening to quickly align the caller and call-taker, communications training for call-takers and public education may support early identification of patients at imminent risk of out-of-hospital cardiac arrest.

## Background

The Chain of Survival in out-of-hospital cardiac arrest (OHCA) illustrates the four key sequential and overlapping steps that effectively optimise survival following OHCA.^1^ The first step in the Chain of Survival is the ‘Early recognition and call for help – to prevent cardiac arrest’. This emphasises the importance of an early call for help for those people at high risk of imminent OHCA^2^, so that Emergency Medical (Ambulance) Service (EMS) staff can be dispatched to arrive as quickly as possible. EMS call-takers have a critical role in this process and have been referred to as the “front line” of EMS.^3^ The majority of the focus in the literature has been on recognising patients who have already suffered an OHCA at the time of the EMS call.^4^ The 2021 European Resuscitation Guidelines^1^ specifically reference prodromal symptoms and specify the importance of recognising patients at imminent risk of OHCA. An example would be the early recognition of patients presenting with chest pain of cardiac origin who are at high risk of collapse. Early recognition would ensure quicker dispatch of EMS and improve likelihood of survival.

National Health Service (NHS) England’s Ambulance Response Programme^5^ explored strategies to reduce operational inefficiencies and improve the quality of care for patients, their relatives and carers. A set of pre-triage questions were introduced which predominantly focussed on assessing consciousness and breathing at the very start of the EMS call. The aim of the pre-triage questions were to support the identification of patients with immediately life-threatening presentations so that an appropriate resource could be dispatched straightaway. Patients who are at imminent risk of OHCA are clearly a key target group for this initiative.

Analysis of caller and call-taker interactions during EMS calls provides an opportunity to understand typical communication behaviours; this could inform developments that lead to earlier and more accurate identification of patients who are at risk of imminent OHCA.^3^ Conversation analysis is considered an “observational science,” focussed on recordings and detailed transcripts of naturalistic talk data. Although a qualitative research method, it is unique as the focus is not participants’ experiences, but rather what participants in an encounter are doing or achieving in, and through, their talk.^6^ There is a growing body of work where conversation analysis has been used to investigate healthcare encounters and conversation analysis research has been successful in investigating the rules and norms that are adopted during routine healthcare tasks.^7^ A recent literature review investigating conversation analysis in emergency settings located a number of studies investigating call openings in EMS calls. Research commonly investigated the more traditional sequences of an EMS call, the greeting sequence, address sequence, and reason-for-the-call sequence.^8^ Our research investigates a corpus of EMS calls where the patient was alive at the initiation of the EMS call, but who subsequently deteriorated into OHCA during the prehospital phase of care (i.e. before arrival at hospital). We used conversation-analytic methods to examine in detail the exchange of information between the caller and call-taker during the pre-triage questions in EMS call openings for any recurrent interactional difficulties.

## Methods

### Study design

Conversation analysis was the chosen methodological approach, as it has been successfully used to produce new insight into how certain communication behaviours might be associated with different patient outcomes during healthcare encounters.^9^

### Ethics

Ethics committee approval was obtained from the University of the West of England (UWE), Bristol (UWE reference: HAS.20.05.182). Health Research Authority approval (19/HRA/4437) and Research and Development Approval from South Western Ambulance Service NHS Foundation Trust were also secured.

### Setting

**Fig. 1:**
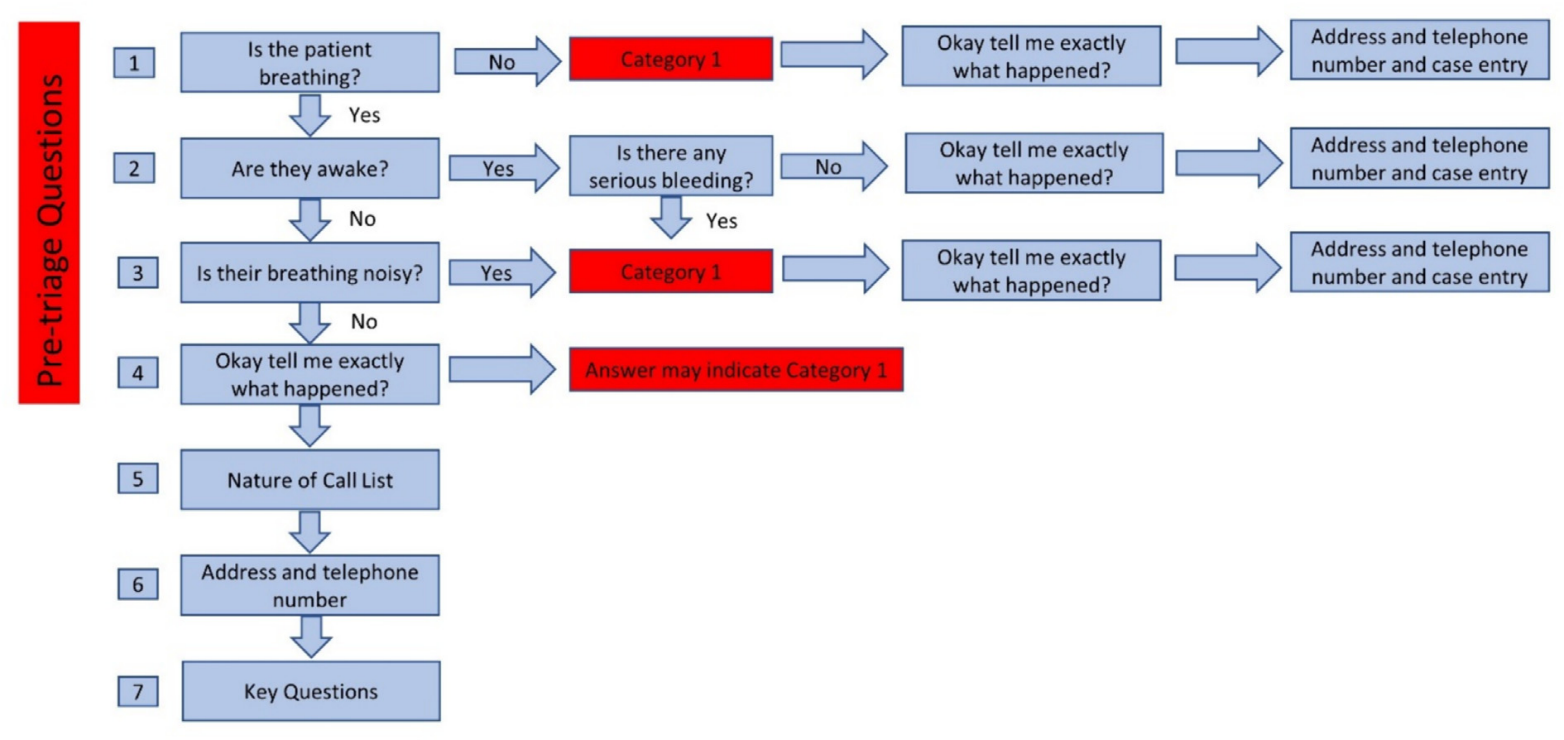
Pre-Triage Questions.

This research was completed in one United Kingdom Emergency Medical (ambulance) Service (EMS) in the South West of England which covers an area of 20,000 square miles and serves a resident population of 5.5 million people.^10^ In this service, non-clinically trained call-takers use Medical Priority Dispatch System (MPDS)^11^ to triage emergency calls. MPDS is a scripted protocol guiding the call-taker through key questions regarding the patient’s condition.^12^ In England, MPDS is prefaced with pre-triage questions illustrated in Fig. 1 and consisting of 3 key questions; Is the patient breathing?; Are they awake?; Is their breathing noisy? Fig. 1 indicates progress through the questions depending on the answers given. It should be noted that the awake and breathing questions may not be revisited after the pre-triage question sequence has been answered.

### Participants and study data

EMS calls made during 2018 and 2019 were sampled from the OHCA registry of the participating EMS. This registry is not publicly available, but maintained locally. Where a patient’s OHCA is unwitnessed by EMS, the time of OHCA is often an estimation. To address this, patients who had a time of OHCA greater than three minutes after the EMS call connected were labelled as ‘not in OHCA at the time of the EMS call’, while patients who had a time of OHCA recorded as less than three minutes after the EMS call was connected were grouped as having suffered an ‘OHCA at the time of the EMS call’. The cut-off time of three minutes was chosen because it was judged to have the best chance of allocating patients to the right groups, but also because this time interval had been previously employed during the Ambulance Response Programme’s “dispatch on disposition” protocol where it was the maximum time between call connect and response time clock start.^5^ According to the 2018–2019 OHCA registry data there were 1344 EMS calls where the patient was identified as not in cardiac arrest at the time of EMS call initiation, but who subsequently suffered an OHCA in the prehospital phase of care and for whom call categorisation data was available. From this group of patients, 50 EMS calls were sampled for conversation analysis. Patients were drawn from two cohorts, those who were triaged as requiring the most immediate ambulance response (Category 1), and those who were triaged as requiring a less immediate ambulance response (Categories 2–5), summarised in Table 1.

**Table 1:** Study cohorts.

Cohort 1	Patients not in cardiac arrest at the time of the EMS call, but who suffered an OHCA in the prehospital phase of care and who **coded** initially as a Category 1 call
Cohort 2	Patients not in cardiac arrest at the time of the EMS call, but who suffered an OHCA in the prehospital phase of care and who **did not** code initially as a Category 1 call

**Fig. 2:**
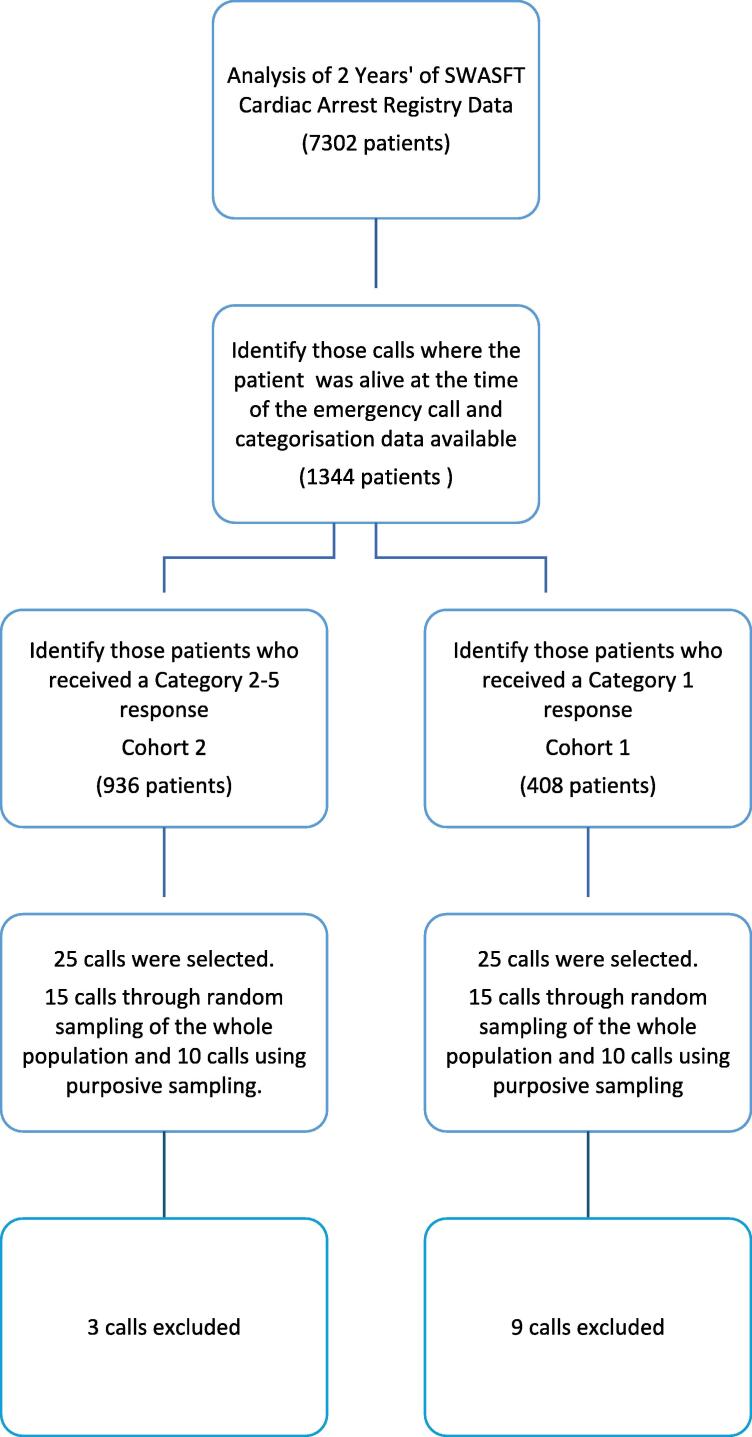
Sampling EMS call data for conversation analysis.

Twenty five EMS calls in each of Cohorts 1 and 2 were selected: 15 calls were selected through random sampling to easily obtain a random subset of calls from the large cohorts. We then used purposive sampling to identify 10 cases of interest^13^ from each cohort. We selected cases of interest to obtain variation in sampling, to include different OHCA situations and EMS calls where patients may have received suboptimal triage. Cases of interest included incidents that had been upgraded to a higher priority response or downgraded to a lower priority response during the EMS call triage process. Cases were also purposively sampled for EMS-witnessed OHCAs, OHCAs occurring in a healthcare facility, patients aged less than 30 years old, unwitnessed OHCAs, EMS-witnessed ventricular fibrillation OHCAs, OHCAs occurring greater than one hour after the EMS call and calls where the final categorisation was Category 4. The process for identifying these calls is described in Fig. 2 and the detail of the included calls is included in Supplementary File 1.

Once the cases were selected, spoken names, telephone numbers and locations were redacted using Audacity^14^ editing software (Muse Group, Pittsburgh) by an EMS data officer. Call data were transferred to a university-approved transcriber using a secure file upload system. The call recordings were initially transcribed verbatim and then reviewed to identify their general overall structural organisation and specific areas of interest. Twelve calls were excluded before analysis for the reasons shown in Table 2 and 38 calls were included for analysis (See Table 3, Table 4, Table 5, Table 6).

**Table 2:** Excluded calls.

**Reasons for exclusion**
Third party caller (2 calls)
Patient potentially agonal breathing (5 calls)
The patient had already suffered an OHCA at the time of the EMS call (declaration of no signs of life) (3 calls)
Patient was hearing impaired, unable to hear questions (1 call)
Unintended contact with the EMS (1 call)

**Table 3:** Extract 1: Cohort 1, Caller 8 (Category 1).

01 CT	Ambulance Service ().
02 C1 →	Yeah hi there.hhh um (.) I just wondered °>if it was<° possible to get an a:mbulance to [ xxxx ]
03 CT →	[Okay ] (0.3) just bear with me (.) i- sorry > I just need to ask you a few questions. is the patient br[eathing < no:rmally?
04 C1 →	[Yes, Uhm:: (.) e’s got pulmonary (0.3) what’s it called?=hhhh (.) um
05 →	(1.1)
06 C2 →	Uhm (0.2) rheumatoid arthritis and pulmonary lungs,
07 C1 →	Oh he’s got pulmona[ry fiBROSis s[o he’s-
08 CT	[Yeah but right- [Right ↓no:w is he bre[athing normally for him?
09 C1	[>bi-<
10	(0.4)
11 C1	°°y-°° fo:r him yeah_he sort of collapsed on the floor_we’ve managed [to get him on the bed, but th-
12 CT	[Okay (0.3) >all right< (0.2) is he awa:y**k**?
13	(0.4)
14 C1	Uh (.) is he awake? is he awake ((Name))?,
15 C2	Eh- (.) yeah (.) he’s #uh (.) #uhm-=

**Table 4:** Extract 2: Cohort 2, Caller 50 (Category 2).

01 CT	Ambulance is the patient breathing?,
02 →	(0.8)
03 C	.hhh uhh no he’s got (.) blood coming out of his mouth and he’s cllapsed
04 CT	Okay and not breathing (.) is that?
05 →	(0.4)
06 C →	Uhm (.) is he breathing, (1.4) yeah he’s just cllapsed and there’s blood’s coming out of his nos[e?=
07 CT	[Yeah but is- is the patient breething
08 C	IS HE BREATHIN (.) I don't know he’s in the pub I’m out here (0.4) calling you,
09	(0.6)

**Table 5:** Extract 3: Cohort 2, Caller 27 (Category 2).

01 CT	A:mbulance ↑Service?, is the patient breath↑ing?
02 →	(0.7)
03 C →	#Ah- #ah- #e- #e- #e- yeah e’s breathing,
04	(0.4)
05 CT →	°#Ah-° (.) he’s bre:athing?
06 C →	Yes he p(h)assed ou[t,
07 CT →	[Thank you- o[kay is he awa:yk?
08 C →	[So I-
09	(0.4)
10 C	°#Ah-° (0.3) yes just about(.) [yes.
11 CT	[Thank you_is there any serious bleeding?
12	(0.5)
13 C	°#Ah-° °#ah-° °#e-° n::o, (0.4) [°no°
14 CT	[Thank ↑you_what’s the address of the emergencee,

**Table 6:** Extract 4: Cohort 1 Caller 14 (Category 2).

01 CT	Ambulance Service, is the patient breathing normally?
02	(0.6)
03 C →	ah- hou-.hh yes well (0.2) she’s just (.) gone to the-.hhh actually she’s been in bed s:ic**k** all week.hhh an’ she’s just tried to get to the <to:il> let and she’s fallen off the toilet;.h[hh and sh-]
04 CT →	[Is she aw ]ay**k**?
05 C →	Just like.hhh she- she is awa:yk but = tu:h.hhh °nu-° (0.3) you know, (0.3) °n-°[. not- ] not-
06 CT →	[Is there a-]
07	(0.3)
08	Is there any serious ↓bleeding

### Data analysis

The EMS call opening (the pre-triage questions) up until the point that the address details were taken (see Fig. 1) was the stage selected for detailed analysis. The pre-triage questions sequence was considered worthy of exploration for several reasons. It is a relatively new addition to the EMS call structure in England, developed to enable the rapid identification of those patients in OHCA, or who are at immediate risk of OHCA.^15^ In the data, this question sequence was found to be a common source of interactional “trouble” for both the caller and the call-taker.

The pre-triage question section of the call recordings was transcribed in detail according to standard Jeffersonian conventions^16^ in preparation for analysis. A key to Jeffersonian transcription conventions is included as Supplementary File Two. Author KK, with support from author RB, systematically analysed the call data to identify recurrent patterns of interest. Conversation analysis methods were used to elucidate the organisation of the action sequences, design of turns-at-talk and the word choices participants made in delivering and responding to the pre-triage questions during the call opening^17^, with a focus upon how these may help identify a patient at imminent risk of OHCA.

## Results

The pre-triage questions in the call opening are quick fire questions with Yes/No answers, designed to enable the call-taker to swiftly identify critically unwell patients before the longer MPDS triage process begins. Our main observation was that the pre-triage question sequence did not lend itself to efficient and accurate triage of critically unwell patients as intended. In 50% of calls (53% in Cohort 1 and 47% in Cohort 2) the caller gave the reason for the call during the pre-triage questions leading to unintended delays and confusion during the triage process. The Category 1 cohort were often triaged as presenting with a time critical breathing presentation in comparison to the Category 2 cohort who appeared to present as less critically unwell. We did not find specific interactional differences between the two cohorts, rather that there were interactional issues that applied to both cohorts. We have set out four separate extracts of call openings to illustrate our findings. Extracts were chosen as they clearly demonstrated interactional trouble.

### A clash of agendas

In our dataset, the caller and call-taker appear to come to the call with different agendas or concerns. The caller is concerned with requesting an ambulance on behalf of the patient as quickly as possible and is unaware of the interactional constraints of the EMS call. The call-taker is under pressure to proceed through the triage process as quickly as possible whilst complying with the script.

In Extract 1, there are two callers, Caller 1 is speaking directly to the call-taker and Caller 2 is in the background. Following the call-taker’s institutional identification at line 1, Caller 1 initiates a request for help as per usual caller expectation for EMS calls.^18^ In initiating the request for help, the caller is hijacking the dispatch protocol^19^ by interjecting before the call-taker can ask the first pre-triage question and this action highlights the clash of agendas between the caller and the call-taker. The call-taker interrupts the caller at line 3, when their turn is clearly in progress (as opposed to about to start or almost hearably complete)^20^. The caller temporarily abandons their request for help instead of continuing and “fighting for the floor”.^21^ The call-taker continues on at line 3 with the first breathing pre-triage question. Lines 4 to 7 are where the caller answers more than the question, using narrative expansion. At line 11, the caller attempts to initiate the reason for the call, and appears to be on the way to informing the call-taker that there is an issue when the call-taker interrupts them again to ask the second pre-triage question, ‘Is he awake?’. This interruption is a consequence of the call-taker orientating to their own concerns − institutional tasks that need to be accomplished (the pre-triage question sequence). There are therefore delays in progression of the call caused by nonconformity (jeopardising interaction progression) in the interaction as both parties persist in pursuing their own agenda.

In Extract 2, the caller cannot answer the pre-triage questions as they are not with the patient, but they continue to pursue their own agenda of giving information and getting an ambulance to attend as quickly as possible. There are significant inter-turn delays at lines 2 and 5 indicating interactional trouble and an intra-turn delay in line 6 when pushed to answer if the patient is breathing. The call-taker clearly notices this uncertainty and pursues the response for a second time at which point the caller admits that they do not know the answer.

In Extract 3 there is a delay in responding to the breathing pre-triage question. The delay which can hint at difficulty in responding is marked by a gap at line 2 and the non-lexical vocalisations uttered before giving an answer in line 3. This delay in response might be due to a lack of awareness on the caller’s part of the structure of the EMS call, the desire to give the reason for the call, or there could be an issue with the patient’s breathing. Here the call-taker does not immediately accept the caller’s answer to the breathing pre-triage question as evidenced at line 5 by them issuing a question for confirmation. This recognition of interactional trouble by the call-taker is important as the interactional pattern can indicate abnormality in breathing and the possibility that the patient is deteriorating, and this can be acted on later in the call. At line 6 the caller offers information concerning the reason for the call before they are asked. This is overlapped at line 7 for the second ‘awake’ pre-triage question by the call-taker. There is an overlap by the caller at line 8 where you can assume they want to give the reason for the call and then a difficulty in responding to the final pre-triage question.

### Narrative expansions

Another way that callers pursue their agenda is to initially offer a conforming, but minimal, answering response to the first pre-triage question and then expand it, answering ‘more than the question,’ with a narrative concerning their reason for the call^22^. Within the dataset there were three examples where the caller gives a minimal response plus a narrative expansion to the breathing pre-triage question (Calls 8 (Extract 1), 14 and 32). These callers interactionally depart from the agenda of the call-taker’s question by introducing the reason for their call at the first opportunity. An example is illustrated in Extract 4 below where the call was initially coded as Category 2.

In Extract 4, after a 0.6 s delay in responding to the call-taker’s breathing pre-triage question, and some perturbation, in line 3 the caller replies with “yes” and begins their next turn-at-talk with the word “well” before elaborating. Using “well” as a preface to a turn-at-talk can indicate non-straightforwardness in responding.^23^ However, the call-taker appears to accept this “yes” as a straightforward confirmation that the patient is breathing normally, evidenced by their proceeding to ask the next question at line 4. At line 5, the caller answers the awake pre-triage question, going on to qualify their response and the call-taker interrupts their qualification at line 6.

The effect of the narrative expansion impacts the critical pre-triage question sequence. It delays progressivity and the call-taker can easily interpret the initial “yes” as straightforward confirmation that the patient is breathing, or breathing normally. Important signals that impact on the call-taker’s understanding of the patient’s situation may be lost as there may be no repeat of a breathing assessment.

## Discussion

Our analysis of a sample of EMS call openings for patients who are at imminent risk of OHCA indicates that the call opening and the pre-triage question sequence do not lend themselves to an efficient exchange of information as intended. The design of the call opening leads to a clash of agendas between the caller and call-taker leading to interactional trouble that both parties to the call have to work to overcome. This interactional trouble results in time delays and information loss. The caller and call-taker are not enabled to efficiently work together to complete the call triage accurately and efficiently.

Whilst the focus of our study was patients at imminent risk of OHCA our research findings are likely to apply to all EMS calls, regardless of the presenting complaint. We examined two cohorts of calls, with purposive sampling to increase variation in cases, but the interactional issues identified were present in both cohorts. Our findings are particularly important for patients at imminent risk of OHCA. Early recognition of this patient group means that interventions such as community first responder attendance, early access to a community defibrillator, a call-taker staying on the call to provide timely telephone cardiopulmonary resuscitation (CPR) advice, and an EMS resource dispatched at the highest priority can be actioned to improve survival.^26^ The immediate initiation of CPR from a community first responder, bystander or trained professional can double or quadruple survival from OHCA.^27^

The EMS call is an example of institutional talk (e.g., medical interaction). Institutional talk is different to ordinary conversation in that it is designed to serve an institutional agenda. Heritage^24^ describes ordinary conversation as the ‘master institution’ with institutional talk as a ‘restricted local variant’ in which specific and distinct tasks are addressed in a particular way. This is the case in the EMS call data where turns at talk are ordered via question–answer sequences across a number of activity phases or tasks to be achieved, incrementally moving towards the end goal of completion. EMS calls are monotopical and time-sensitive – questions are designed to elicit information regarding the seriousness of the situation as efficiently as possible so that the call can be triaged and the correct response category allocated to the emergency.^25^

One major feature of this EMS call data is that the call opening (i.e. the pre-triage questions) are scripted and call-takers have little flexibility to deviate from the script. Calling an ambulance is not common practice and the average person will only contact EMS twice in their lifetime.^28^ The call-taker will be experienced in the design of the institutional interaction, but the caller is likely inexperienced, with heightened emotions^24^ and these factors contribute to dysfunction in the exchange of information.

The call-taker and caller come to the EMS call with distinct goal orientations (e.g. call-takers treat the callers as routine cases and conversely the caller sees their case as unique and personal^29^; the caller and call-taker work together across the different activity phases in ways that co-construct it as an EMS call despite pursuing their own agenda or concerns. Traditionally, calls for help allocate the first topic slot to the caller and it is here where the ‘reason for the call’ is delivered regardless of the call-taker’s opening sequence.^18^ In our corpus of calls, the callers’ expected sequence of events does not occur because the pre-triage question sequence is launched immediately at the call opening, disrupting the caller’s expectations.

In our related research, call-takers have stressed that the caller does not expect to be asked a question about breathing immediately during call opening and callers often miss the initial pre-triage question completely.^30^ The caller persists with the “what” and the “where” because they are following more usual call conventions. Riou et al.^19^ identified that in cases where callers give the reason for the call before they are asked for it (pre-emption), when they are asked for the reason for the call later in the script, the caller treats this as a request for more information and original information regarding the reason for the call is lost. The caller does not repeat what has already been said and this leads to miscommunication creating inefficiency and the risk of information loss with associated delays in providing appropriate assistance.

The pre-triage questions were introduced in England at the point of EMS call connection and before the call-taker proceeded through the main MPDS triage, in order to quickly identify those patients that require the highest priority ambulance response. An additional element of the Ambulance Response Programme was allowing call-takers more time (240 s in total) to triage calls so that more accurate triage could be achieved, with the aim of providing a more appropriate ambulance response to patients. The introduction of the pre-triage questions provided assurance that with the longer triage time, EMS could quickly identify the patients requiring a fast response and dispatch within 30 s of call connection; these target patients included those already in OHCA and those at imminent risk of OHCA. The Ambulance Response Programme found that the pre-triage questions supported identification of 75% of patients requiring an immediate ambulance response within 30 s.^31^ However this leaves 25% not identified within 30 s. We have focused our research on patients at imminent risk of OHCA, a high risk patient group who require immediate intervention. We have highlighted the interactional challenges with the EMS call opening that cause delay to treatment. Improvements to the call opening and pre-triage question sequence could be designed and tested with the aim of supporting fast identification and optimal dispatch.

Similar to the pre-triage question sequence intervention, a team from Denmark initiated a quality improvement programme to improve the recognition of OHCA patients during EMS call triage. An element of the programme was a No-No-Go algorithm asking:- is the patient conscious? Is the patient breathing normally? If the answer to both of these questions was “No” then the call-taker began CPR instructions. The study included 209 patients post-implementation and concluded no improvement in the time to asking essential questions after the implementation of the No-No-Go algorithm.^32^ Call-takers could choose not to use the No-No-Go algorithm and it would be a useful addition to the literature to explore with call-takers the reasons why the implementation algorithm did not reduce the time to asking essential questions for patients in OHCA.

Improvements to the EMS call opening could include adjustments to the design of the call-opening to quickly align the caller and call-taker, though the effect of this on all calls would need to be assessed. A quick greeting by the call-taker and a very brief explanation that the call-taker will ask a series of questions, stressing the importance of quick and accurate answers may be helpful. Our previous research investigating call-taker views on identifying patients at imminent risk of OHCA has already highlighted that they desire redress of the imbalance of call-taker training on IT systems over communications training.^30^ Education of members of the public regarding the structure of the EMS call is important and we need to understand how this can be achieved.

Further research is needed to understand how modifications to the EMS call opening could enable fast orientation and prevent interactional challenges that delay progression and subsequent dispatch. Other studies could explore how best to enhance communications training to support call-takers to manage the interaction efficiently; and how to effectively educate members of the public regarding the interactional sequence of an EMS call.

### Limitations

The data for this study were collected in one English EMS and this service uses pre-triage questions and MPDS. The call-takers are not clinically trained, and the findings may not be generalisable to other EMS settings using alternative systems.

## Conclusions

In this dataset, the pre-triage questions in the EMS call opening cause interactional trouble that impacts on call progression and risks critical information loss. This finding is unlikely to be limited to EMS calls regarding patients at imminent risk of OHCA. The gains from improving the efficiency of the EMS interaction will be greatest for those patients who will benefit the most from early intervention, as is the case for patients at imminent risk of OHCA. There are opportunities to improve the recognition of patients that are at imminent risk of OHCA so that they can receive the fastest possible (Category 1) response. It is feasible that these findings could be used to support modifications to the EMS call opening and communication-based training for call-takers. Public education on the structure of EMS calls would help to manage caller expectations and optimise the EMS call process.

## Funding

Dr Kim Kirby completed this work during a Clinical Doctoral Research Fellowship funded by National Institute for Health and Care Research in the UK (ICA-CDRF-2018-04-ST2-007).

## CRediT authorship contribution statement

**Kim Kirby:** Writing – review & editing, Writing – original draft, Methodology, Investigation, Funding acquisition, Formal analysis, Data curation, Conceptualization. **Sarah Voss:** Writing – review & editing, Writing – original draft, Supervision, Conceptualization. **Jonathan Benger:** Writing – review & editing, Writing – original draft, Supervision. **Rebecca K. Barnes:** Writing – review & editing, Writing – original draft, Supervision, Methodology, Investigation, Formal analysis, Conceptualization.

## Declaration of competing interest

The authors declare that they have no known competing financial interests or personal relationships that could have appeared to influence the work reported in this paper.
