# Supplementary material for: A conversation analytical study of call openings in Emergency Medical Service calls where the patient is at imminent risk of out-of-hospital cardiac arrest
Source: Resusc Plus. 2024 Jul 5;19:100706. doi: 10.1016/j.resplu.2024.100706 (PMC11403134; doi:10.1016/j.resplu.2024.100706)
Supplement: Supplementary Data 1 [file mmc1.docx]

**Details of included calls**

*Highlighted bold text indicates change in categorisation

| **Call** | **Patient age** | **Patient sex** | **Caller Status** | **Final category of response** | **Change in category of response** | **Clinical categorisation and any change** | **Call length min:sec** | **Witnessed status** | **Response Time**  **(minutes)**  **Call connect to arrival at scene.** | **Survived to hospital discharge** |
| --- | --- | --- | --- | --- | --- | --- | --- | --- | --- | --- |
| 4 | 44 | Male | Family/  friend | Cat 1 | Cat2-Cat1 | Not alert with Chest Pains to Cardiac  **→Respiratory Arrest – breathing Uncertain (Agonal)** | 11:51 | Bystander | 9 | No |
| 6 | 65 | Male | Family/  friend | Cat 1 | Cat2-Cat1 | Unconscious or Fainting – Effective Breathing  **→Respiratory Arrest – Ineffective Breathing** | 11:11 | Bystander | 9 | No |
| 7 | 60 | Female | Family/  friend | Cat 1 | Cat2-Cat1 | Difficulty speaking between breaths **→ known lung disorder with ineffective breathing** | 11:22 | EMS | 9 | No |
| 8 | 71 | Male | Family/  friend | Cat 1 | Nil | Ineffective breathing  **→Cardiac/Respiratory Arrest – Breathing** **Uncertain (Agonal)** | 20:36 | Bystander | 17 | No |
| 11 | 65 | Male | Family/  friend | Cat 1 | Nil | Ineffective breathing | 3:52 | EMS | 10 | No |
| 12 | 92 | Male | Family/  friend | Cat 1 | Nil | Ineffective breathing  **→Cardiac/Respiratory Arrest – Breathing Uncertain (Agonal)** | 11:40 | EMS | 10 | No |
| 14 | 80 | Female | Family/  friend | Cat 1 | Cat2-Cat1 | Not alert after falling  **→Cardiac / Respiratory Arrest - Not Breathing at all** | 5:18 | Bystander | 33 | No |
| 15 | 91 | Male | Bystander | Cat 1 | Nil | Cardiac / Respiratory Arrest - Breathing Uncertain (Agonal)  **→Drowning / Diving /SCUBA Accident - Underwater domestic rescue** | 8:19 | Bystander | 4 | No |
| 16 | 77 | Male | Bystander | Cat 1 | Cat 2-Cat 1 | Unconscious or Fainting - Not Alert  **→ Respiratory Arrest - Ineffective Breathing** | 27:30 | Bystander | 26 | No |
| 17 | 69 | Male | Family/  friend | Cat 1 | Cat 2-Cat 1 | Unconscious or Fainting - Not Alert  **→ Unconscious Agonal / Ineffective Breathing** | 10:55 | Bystander | 7 | No |
| 18 | 49 | Female | Family/  friend | Cat 1 | Cat 3 – Cat 1 | Sick Person Abnormal breathing  **→ Not Alert and Haemorrhaging Medical Bleed** | 30:36 | Bystander | 9 | No |
| 19 | 71 | Female | Family/  friend | Cat 1 | Cat 4-Cat 1 | Stroke with Abnormal Breathing No evidence (Less than X hrs) – passed for clinical review | 7:08 | EMS | 48 | No |
| 20 | 67 | Female | Family/  friend | Cat 1 | Cat 5-Cat 1 | Fallen over 6hrs with Injuries to proximal area without priority symptoms  **→ Unconscious post fall** | 5:11 | Bystander | 15 | No |
| 21 | 57 | Male | Family/  friend | Cat 1 | Nil | Unconscious post fall | 9:16 | EMS | 6 | No |
| 22 | 50 | Male | Healthcare Professional | Cat 1 | Nil | Cardiac / Respiratory Arrest - Not Breathing at all | 9:20 | EMS | 6 | No |
| 23 | 18 | Female | Bystander | Cat1 | Nil | Continuous or Multiple Fitting  **→ Fitting and Not Breathing** | 8:12 | EMS | 4 | No |
| 26 | 70 | Male | Family/  friend | Cat 2 | Nil | Chest Pains Heart attack or Angina History | 5:07 | EMS | 2 | No |
| 27 | 77 | Male | Family/  friend | Cat 2 | Nil | Not Alert after Falling  **→ Not Alert after Falling - Still on the Ground** | 3:54 | EMS | 8 | Yes |
| 28 | 80 | Male | Patient (first party caller) | Cat 2 | Nil | Difficulty speaking between breaths | 2:23 | Bystander | 38 | No |
| 29 | 60 | Male | Family/  friend | Cat 2 | Nil | Unconscious or Fainting - Effective Breathing | 9:46 | EMS | 6 | No |
| 30 | 63 | Male | Family/  friend | Cat 2 | Nil | Difficulty speaking between breaths | 3:22 | Bystander | 18 | No |
| 31 | 32 | Male | Family/  friend | Cat2 | Nil | Overdose/Poisoning (ingestion) Not Alert  **→Intentional Overdosed and Not Alert** | 2:28 | Bystander | 69 | No |
| 32 | 59 | Female | Family/  friend | Cat 2 | Nil | Not Alert with Breathing Problems  **→ Stroke Not Alert No evidence (Less than X hrs)** | 8:50 | Bystander | 42 | No |
| 33 | 45 | Male | Family/  friend | Cat3 | Nil | Chest Pain over 35 breathing Normally | 3:32 | EMS | 43 | Yes |
| 34 | 55 | Male | Family/  friend | Cat2 | Nil | Difficulty speaking between breaths | 8:18 | EMS | 4 | Yes |
| 35 | 79 | Female | Family/  friend | Cat 2 | Nil | Not Alert after Falling | 7:20 | Bystander | 10 | No |
| 36 | 84 | Male | Family/  friend | Cat 2 | Cat4-Cat2 | Fallen - Not Dangerous Proximal Area Injuries  **→Difficulty speaking between breaths** | 6:18 | EMS | 9 | No |
| 38a | 31 | Male | Family/  friend | Cat 2 | Cat5-Cat2 | Abnormal Breathing | 1:24 | EMS | Unknown | No |
| 38b | 31 | Male | Family/  friend | Cat 2 | Cat5-Cat2 | Abnormal Breathing | 8:04 | EMS | Unknown | No |
| 40 | 78 | Female | Patient (first party caller) | Cat 2 | Nil | Difficulty speaking between breaths | 16:19 | EMS | 15 | No |
| 42 | 76 | Male | Family/  friend | Cat 2 | Nil | Difficulty speaking between breaths | 3:16 | EMS | 13 | Yes |
| 43 | 54 | Male | Family/  friend | Cat 2 | Nil | Clammy with Chest Pains | 5:29 | EMS | 12 | Yes |
| 44 | 91 | Female | Family/  friend | Cat 4 | Nil | Fallen - Not Dangerous Proximal Area Injuries | 3:20 | EMS | 69 | No |
| 45 | 83 | Female | Healthcare Facility | Cat 2 | Nil | HCP & AED On scene (immediately life threatening) A&E transport | 4.50 | Healthcare Professional | 28 | No |
| 46 | 21 | Female | Bystander | Cat 2 | Cat3-Cat2 | Unconscious or Fainting - Alert with Abnormal Breathing  **→Fitting during Pregnancy** | 5.31 | Bystander | 33 | No |
| 47 | 26 | Male | Family/  friend | Cat 2 | Nil | Overdose/Poisoning (ingestion) Not Alert  **→Intentional Overdosed and Not Alert** | 5.29 | EMS | 3 | No |
| 48 | 76 | Female | Healthcare Professional | Cat 2 | Cat4-Cat2 | Female over 45 with Abdominal Pain Above Naval  **→Unconscious or Fainting - Changing Colour** | 4:17 | Unwitnessed | 15 | No |
| 49 | 80 | Female | Bystander | Cat 2 | Cat1-Cat2 | Unconscious post fall | 7:16 | Bystander | 17 | No |
| 50 | 57 | Male | Bystander | Cat 2 | Cat2-Cat1 | Cardiac / Respiratory Arrest - Not Breathing at all  **→Unconscious or Fainting - Not Alert** | 6:49 | Bystander | 8 | No |
