# Supplementary material for: A conversation analytical study of call openings in Emergency Medical Service calls where the patient is at imminent risk of out-of-hospital cardiac arrest
Source: Resusc Plus. 2024 Jul 5;19:100706. doi: 10.1016/j.resplu.2024.100706 (PMC11403134; doi:10.1016/j.resplu.2024.100706)
Supplement: Supplementary Data 3 [file mmc3.docx]

**Key to transcription conventions**

CT = Call taker C = Caller

| (.) | Notable pause but of no significant length |
| --- | --- |
| (0.2) | A timed pause of 0.2 seconds |
| [ ] | A point where overlapping speech occurs |
| > < | Arrows surrounding talk shows that the pace of the speech has quickened |
| < > | Arrows in this direction show that the pace of the speech has slowed down |
| ( ) | Space between brackets denotes that the words spoken here were too unclear to transcribe |
| (( )) | Where double brackets appear with a description inserted it denotes some contextual information |
| What | When a word or part of a word is underlined it signifies emphasis |
| . | Falling or final intonation |
| , | Slightly rising intonation |
| ? | Strongly rising intonation |
| ↑ | Rise in pitch |
| ↓ | Fall in pitch |
| → | Denotes a particular sentence of interest to the analyst |
| HELLO | Capitals indicates something was said loudly, or shouted |
| Rea(h)lly | A bracketed ‘h’ means that there was laughter within the talk |
| = | Represents latched speech, a continuation of talk |
| :: | Represent elongated speech, a stretched sound |
| away**k** | Boldface consonant represents a hardened sound |
